# Relationships between cognitive biases, decision-making, and delusions

**DOI:** 10.1038/s41598-023-36526-1

**Published:** 2023-06-10

**Authors:** Julia M. Sheffield, Ryan Smith, Praveen Suthaharan, Pantelis Leptourgos, Philip R. Corlett

**Affiliations:** 1 Department of Psychiatry and Behavioral Sciences, Vanderbilt University Medical Center, 1601 23rd Ave S, Nashville, TN 37209 USA; 2 Laureate Institute for Brain Research, Tulsa, USA; 3 Department of Psychiatry, Yale University, New Haven, USA; 4 University of Lille, Lille, France

**Keywords:** Human behaviour, Learning and memory

## Abstract

Multiple measures of decision-making under uncertainty (e.g. jumping to conclusions (JTC), bias against disconfirmatory evidence (BADE), win-switch behavior, random exploration) have been associated with delusional thinking in independent studies. Yet, it is unknown whether these variables explain shared or unique variance in delusional thinking, and whether these relationships are specific to paranoia or delusional ideation more broadly. Additionally, the underlying computational mechanisms require further investigation. To investigate these questions, task and self-report data were collected in 88 individuals (46 healthy controls, 42 schizophrenia-spectrum) and included measures of cognitive biases and behavior on probabilistic reversal learning and explore/exploit tasks. Of those, only win-switch rate significantly differed between groups. In regression, reversal learning performance, random exploration, and poor evidence integration during BADE showed significant, independent associations with paranoia. Only self-reported JTC was associated with delusional ideation, controlling for paranoia. Computational parameters increased the proportion of variance explained in paranoia. Overall, decision-making influenced by strong volatility and variability is specifically associated with paranoia, whereas self-reported hasty decision-making is specifically associated with other themes of delusional ideation. These aspects of decision-making under uncertainty may therefore represent distinct cognitive processes that, together, have the potential to worsen delusional thinking across the psychosis spectrum.

## Introduction

Delusions are unfounded and firmly held beliefs that disrupt social functioning and psychological well-being^1,2^. Although delusions are a hallmark symptom of psychotic disorders, there is increasing evidence that delusional thinking exists on a continuum of severity, impacting quality of life and behavior in a wide range of individuals^3^. How and why delusional thinking manifests are critical questions for understanding prevention and intervention. One important set of cognitive mechanisms underlying delusions may involve decision-making, particularly in light of new or changing information. Knowing how different aspects of decision-making intercorrelate and uniquely relate to delusional thinking may support better cognitive models of delusions. These models can ultimately be used for designing specific interventions, in order to test causality of altered decision-making processes in the formation and maintenance of delusions^4^.

To date, the role of cognitive biases in delusional ideation has received most scrutiny. A prominent example is jumping to conclusions (JTC)—the tendency to make hasty decisions based on a relatively small amount of information. The JTC bias can be measured using a behavioral task^5,6^ in which the tendency of individuals to make a decision after only one or two observations has been associated with delusional thinking^7^. Bayesian modeling of this behavior has suggested that it reflects a bias of the individual’s decision-making towards newly presented information^8^, possibly due to noisier or less precise prior beliefs about the environment^9–12^. Yet, JTC behavior as measured on tasks has also been found to be sensitive to confounding processes, such as lower IQ^13^ and the incentives of the task^14^. An alternative assessment of the JTC cognitive bias is through self-report. Questionnaires such as the Davos assessment of cognitive biases scale (DACOBS)^15^) measure an individuals’ self-reported propensity towards hasty decision-making (e.g. “I quickly find evidence to support my beliefs” and “I don’t need all the facts to reach a conclusions”). Prior work has shown some convergence of self-reported and task-based JTC^15^ and specific relationships between self-reported JTC and delusion conviction on clinical interview^16^, suggesting sensitivity to relationships with clinical phenomena. In fact, examination of both task-based and self-reported JTC revealed that self-reported JTC, but not task-based JTC, was significantly associated with positive symptoms in schizophrenia assessed via clinical interview. This relationship remained when controlling for neuropsychological performance, whereas task-based JTC only showed significant relationships with measures of attention and working memory^17^. Therefore, although JTC tasks can measure decision-making, self-reported JTC appears to be an effective way of examining how one’s own sense of their hasty decision making relates to clinical phenomena; however, its role in delusional thinking, and independence from other variables indexing cognitive biases and decision-making, requires continued investigation.

The bias against discomfirmatory evidence (BADE) is another cognitive bias that reflects a failure to revise one’s beliefs about something in light of new evidence to the contrary. If JTC leads to hasty inference, BADE might maintain that new belief over time. Meta-analyses have confirmed elevated BADE in delusional individuals^18^, though it is by no means present in all people with delusions, and its operation may rule out particular accounts of how delusions form^19^. BADE is often measured using an objective vignette-based task^20^, in which participants are presented with a scenario and asked to rate the plausibility of different explanations of the events within it. Participants are then presented with new information regarding the scenario and need to rerate how they perceive each explanations’ plausibility. Scoring of the BADE task varies, but recent methods have identified evidence integration impairment (EII) as significantly associated with overall delusional ideation in non-clinical individuals from the general population^21^. EII measures how strongly the individual endorses implausible scenarios, such that greater BADE-EII reflects a stronger reliance on previously formed beliefs in light of new contradictory evidence.

Cognitive insight involves the self-reported ability to balance self-reflectiveness and self-certainty. Cognitive insight is measured using the Beck Cognitive Insight Scale (BCIS), which provides a composite score defined by the difference between self-reported self-reflectiveness and self-certainty^22^. Those with “healthier” thinking tend to be more reflective and open to feedback, with lower rigidity in their beliefs. On the other hand, individuals with a psychotic disorder who endorse delusional thinking through clinical interview show less willingness to reflect on alternative explanations (low self-reflectiveness) and greater overconfidence (self-certainty). Lower cognitive insight indicates overconfidence in decision-making^23^. Reduced cognitive insight has been associated with delusions^24^ and change in delusional conviction during a clinical trial is associated with improvements in self-certainty^25^.

Despite decades of research on the impact of cognitive biases on delusional thinking, and targeted psychotherapy interventions designed to treat them^26,27^, their ability to explain delusion severity remains limited^28^. Recently, decision-making behavior observed during probabilistic reversal learning and explore/exploit tasks have been associated with delusional thinking^29,30^ and may therefore explain other aspects of decision-making relevant to unusual beliefs. These aspects include a sensitivity to unpredictability, which can be observed during probabilistic reversal learning tasks, and altered rates of belief updating, which can be observed during explore/exploit tasks. For reversal learning, win-switch rate (i.e., the propensity to change responses after a win) and prior beliefs about environmental volatility (i.e., the belief that the task environment is frequently changing) have been associated with paranoia in both individuals with schizophrenia and in the general population^30–32^. These aspects of decision-making are related, as maladaptively high levels of win-switching may be influenced by a stronger belief that the task environment is unstable and frequently changing. It may be that jumping to conlusions bias and win-switching are related via the same volatility mis-estimation mechanism^9,10,19^.

During an explore/exploit task, individuals are presented with options and must decide whether to exploit an option that has already shown evidence of greater reward or explore a second option that may (or may not) turn out to be more rewarding. Evidence suggests that people tend to use a combination of directed and random exploration, in order to maximize reward^33^. Directed exploration requires more cognitive resources to weigh the pros/cons of each option and corresponding expected values. Random exploration (exploring by chance) still allows for information gathering and uncertainty reduction without being so cognitively taxing, however is driven by decision noise, making it potentially less effective as an overarching strategy. Recent work in schizophrenia revealed that greater random exploration was associated with greater overall positive symptom severity^29^, suggesting for the first time that patients with more psychotic symptoms exhibit more behavioral variability during explore/exploit decisions.

Both prior beliefs about volatility and random exploration are computationally derived parameters that take into account trial-by-trial decision-making. We have yet to clarify whether such computational parameters estimated from decision-making tasks explain more variance in delusional thinking than “standard” summary measures (e.g., win-switch rate). Nor do we know whether distinct computational measures derived from different tasks and models are related, and explain shared or unique variance in relationship to delusions.

Finally, delusions tend to focus on certain themes of unusual thinking, including persecution, grandiosity, alien control phenomena, or somatic concern. These might manifest as believing others are plotting against you, that have special powers or abilities, your thoughts can be accessed by others, or that something is wrong with your body that you can’t explain. Persecution is the most common^3^, manifest in the general population as paranoia, and in the clinical extreme, as persecutory delusion. Yet, the specific cognitive mechanisms that contribute to different types of delusions are still under investigation. Recently, reversal learning data from the current dataset were found to be specifically associated with paranoia, but not unusual thought content^30^. Whether other decision-making mechanisms are common to both paranoia and other themes of unusual thinking (hereafter called ‘delusional ideation), or distinct, remains unclear.

Together, the above literature suggests that multiple aspects of decision-making contribute to delusional thinking. Yet, their inter-correlation and specificity has not before been investigated. Here, we aim to extend prior work in this dataset examining specific associations between reversal learning metrics and paranoia^30^. We assess relationships between multiple cognitive biases and decision-making metrics in individuals with schizophrenia and healthy comparison participants and test their joint contribution to variance in paranoia and delusional ideation. Specifically, we assess whether self-reported JTC using the DACOBS^15^, BADE-EII using the BADE task^20^, self-reported BCIS^22^, win-switch rate (WSR) and prior on volatility ($\mu_{3}^{0}$) from a probabilistic reversal learning task^32^, and random exploration (unequal and equal) from an explore/exploit task^33^, are significantly associated with self-reported paranoia (revised Green Paranoid Thoughts Scale-b (r-GPTSb))^34^, self-reported delusional ideation *Peters Delusions Inventory (PDI-21)^35^, clinically-rated suspiciousness/persecution (Positive and Negative Syndrome Scale (PANSS) P6 item) or clinically-rated delusions (PANSS-P1 item)^36^. Self-reported cognitive biases and task-based measures are included in the same model, to determine their specific associations with paranoia and delusional ideation. In addition, computationally-derived task-based measures of decision-making ($\mu_{3}^{0}$, random exploration) were included in different models than the behaviorally-derived task-based measure (WSR) for two reasons: (1) $\mu_{3}^{0}$ and WSR are highly inter-related and (2) we wanted to examine whether inclusion of computationally-derived measures explained more variance in delusions than more standard descriptions of behavior.

All analyses control for age and gender. Group is included as a covariate for analyses predicting self-reported paranoia (r-GPTSb) and self-reported delusional ideation (PDI-21), as all participants are included in these analyses. Clinically-rated paranoia (PANSS-P6) and delusions (PANSS-P1) are measured only in the schizophrenia participants.

## Results

### Group differences

Demographic information for participants in the study are presented in Table 1. The final sample for behavioral data included 42 schizophrenia and 45 healthy participants, who were statistically similar on age, gender, race, and parental education. Analyses including random exploration from the explore/exploit task were conducted in the subset of individuals who completed the Horizon task: 32 schizophrenia and 40 healthy participants.

**Table 1:** Cognitive Ability was measured using the Screen of Cognitive impairment in psychiatry (SCIP)

	Controls N = 45	Schizophrenia N = 42	Statistics controls versus schizophrenia
Age	30.1 (7.86)	27.4 (5.68)	t(85) = 1.88, *p* = .06
Gender (Male/Female)	28/17	31/11	X^2^(87) = .25, *p* = .26
Race (White/Black/Other)	31/9/5	27/12/2	X^2^(86) = 2.19, *p* = .53
Parental Education	14.74 (2.23)	14.61 (2.8)	t(85) = .25, p = .40
Premorbid IQ	114.7 (7.8)	105.2 (13.23)	t(85) = 4.12, *p* < .001
Cognitive Ability (SCIP)	.49 (.72)	− .45 (.92)	t(85) = 5.32, *p* < .001
R-GPTS-b	1.6 (2.7)	1.7 (3.8)	t(85) = − .10, *p* = .92
PDI-21 Total	12.9 (17.1)	23.9 (31.4)	t(85) = -2.1, *p* = .04
PANSS P1	–	2.4 (1.2)	
PANSS P6	–	2.7 (1.1)	

Cognitive Ability was measured using the *SCIP* Screen of Cognitive impairment in psychiatry, *R-GPTS-b* Revised Green et al. Paranoid thoughts scale, *PANSS* Positive and negative syndrome scale, *PDI-21* Peters delusions inventory-21 item.

Healthy controls and individuals with schizophrenia did not significantly differ on self-reported JTC (U = 845.5, *p* = .40), BADE-EII (U = 979, *p* = .77), BCIS (U = 1098, *p* = .19), directed exploration (U = 524, *p* = .19), random exploration (equal) (U = 468, *p* = .05), random exploration (unequal) (U = 675, *p* = .40), lose-stay rate (U = 719.5, *p* = .06) or $\mu_{3}^{0}$ (prior on volatility) from the reversal learning task (U = 1114, *p* = .15). As previously reported in this dataset, individuals with schizophrenia had significantly elevated WSR during reversal learning (U = 1179.5, *p* = .04). These group differences, as well as directed exploration and learning rate from Horizon task, are presented in Fig. S1.

### Zero-order correlations

Correlations between cognitive variables are presented in Table 2. In the schizophrenia group, the cognitive measures were almost entirely independent from one another. Across the whole sample, the relationships of variables from different tasks that reached uncorrected statistical significance were: random exploration (equal) with WSR (ρ = − .25, *p* = .03) and lose-stay rate (ρ = .35, *p* = .002), and BADE-EII with random exploration (unequal) (ρ = .25, *p* = .04). In healthy controls BADE-EII was associated with random exploration (unequal) (ρ = .41, *p* = .01) and with win-switch rate (ρ = .32, *p* = .03). No relationships survived multiple comparisons correction for all tests performed .

**Table 2:** a: All participants, Spearman’s ρ (*p*-value), b: Healthy Controls, Spearman’s ρ (*p*-value), c: Schizophrenia, Spearman’s ρ (*p*-value).

	JTC	BADE EII	BCIS	WSR	LSR	${\varvec{\mu}}_{3}^{0}$	REe	REu	SCIP
All participants, Spearman’s ρ (*p*-value)									
Self-reported JTC	–								
BADE EII	.04	–							
BCIS	− .14	.08	–						
Win-Switch rate	.06	.17	− .10	–					
Lose-stay rate	.13	.18	.02	− .20	–				
Prior on volatility ($\mu_{3}^{0}$)	− .18	.04	− .07	**.54 (< .001)**	− **.26 (.02)**				
Random exploration (equal)	.14	.00	.11	− **.25 (.03)**	**.35 (.002)**	− .24			
Random exploration (unequal)	.23	**.25 (.04)**	.21	.09	.11	.09	.13		
Cognitive ability (SCIP)	− .10	− .14	.03	− **.36 (< .001)**	**.28 (.01)**	− **.23 (.03)**	.09	− .13	

Healthy controls, Spearman’s ρ (*p*-value)									
Self-reported JTC	–								
BADE EII	.02	−							
BCIS Total	− .23	.24	−						
Win-Switch rate	.08	**.32 (.03)**	− .02	–					
Lose-stay rate	.03	.10	.06	− .11	–				
Prior on volatility ($\mu_{3}^{0}$)	− .03	.24	− .15	**.53 (< .001)**	− .08	–			
Random exploration (equal)	.14	− .28	− .09	− .26	.44 (.01)	− .14	–		
Random exploration (unequal)	.21	.**41 (.01)**	.12	.29	.20	.20	.09	–	
Cognitive ability (SCIP)	− .11	− **.41 (.01)**	− .05	− .30	− .13	− .17	.02	− .18	–

Schizophrenia, Spearman’s ρ (*p*-value)									
Self reported JTC	–								
BADE EII	.07	–							
BCIS	− .04	− .04	–						
Win-Switch rate	.08	.11	− .20	–					
Lose-stay rate	.16	.28	.01	− .22	–				
Prior on volatility ($\mu_{3}^{0}$)	− .30	− .11	− .07	**.55 (< .001)**	− **.40 (.01)**	–			
Random exploration (equal)	.14	.33	.32	− .19	.15	− .32	–		
Random exploration (unequal)	.28	.13	.27	− .11	.02	− .11	.24	–	
Cognitive ability (SCIP)	− .23	.04	.28	− **.32 (.04)**	.27	− .18	.01	− .18	–

*BADE EII* Bias against disconfirmatory evidence—Evidence integration impairment, *BCIS* Beck cognitive insight scale, *JTC* Jumping to conclusions, *LSR* Lose-Stay rate, *REe* Random exploration (equal), *Reu* Random exploration (unequal), *SCIP* Screen for cognitive impairment in psychiatry, measuring cognitive ability.

Significant values are in [bold].

### Regression models

Linear regression models were used to examine associations between predictor variables and delusions. In order to determine the best model for predicting delusions, a LASSO regression was conducted first on the full model to aid in variable selection. LASSO ‘shrinks’ coefficients to zero, thereby removing variables from the model, when they are not sufficiently influential^37,38^. Linear regressions were then conducted on the simpler model that remained after the LASSO technique was applied. This allowed us to minimize model over-fitting on our estimates and examine only those variables most meaningfully related to delusions. Linear regressions for ‘behavioral measures’ included age, gender, group, WSR, BADE-EII, self-reported JTC and BCIS as predictors, whereas regressions for ‘computational measures’ included age, gender, group, $\mu_{3}^{0}$, random exploration (unequal), random exploration (equal), BADE-EII, self-reported JTC, and BCIS as predictors. Paranoia (r-GPTSb or PANSS-P6) and delusional ideation (PDI-21 or PANSS-P1) were initially examined as residuals, controlling for one another (e.g. r-GPTSb controlling for PDI-21), to maximize interpretation of specificity with theme of delusional thinking.

Full results from regression models are presented in Table 3.

**Table 3:** Results of Lasso regression.

	PANSS-P6	r-GPTSb	PANSS-P1	PDI-21
Regression with behavioral measures				
Age	–	–	–	–
Gender	0.26	–	–	–
Group	*N/A*	− 0.30	*N/A*	0.46*
WSR	0.29*	0.22*	–	–
BADE-EII	− 0.01	0.01	–	–
Self-reported JTC	–	–	–	0.06**
BCIS	–	− 0.02	–	0.02
Cognitive ability	–	–	–	–
Regression with computational measures				
Age	–	–	–	–
Gender	–	–	–	–
Group	*N/A*	− 0.36	*N/A*	0.46
${{\mu}}_{3}^{0}$	0.52***	0.29**	–	–
BADE-EII		0.01**	–	0.006
Random exploration (Unequal)	0.16*	− 0.07	–	–
Random exploration (Equal)	–	0.04	–	–
Self-reported JTC	–	–	0.09*	0.06*
Age	–	–	–	0.04
Cognitive ability	–	–	–	–

Beta values presented with corresponding significance ***p < .001, **p < .01, *p < .05.

#### Paranoia—controlling for delusional ideation

##### Behavioral measures and self-report

**Figure 1:**
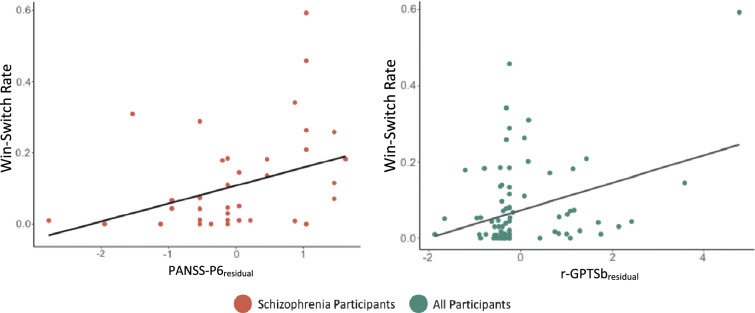
In schizophrenia participants alone (ρ = .35, *p* = .02), and across the whole sample (ρ = .26, *p* = .02), elevated win-switch rate during the 3PRL task was significantly associated with paranoia. Paranoia scores represent residuals controlling for delusional ideation as measured on the PDI-21 or PANSS-P1.

In patients, LASSO regression revealed that gender, WSR and BADE-EII were the best predictors of PANSS-P6, however only WSR ($\beta$ = .29, *p* = .02) was a significant predictor of PANSS-P6, explaining 13% of the variance (R^2^ = .13, *p* = .02) (Fig. 1). Across the whole sample, the best model included group, WSR, BADE-EII and BCIS. Again, only WSR ($\beta$ = .23, *p* = .02) was significantly associated with paranoia as measured by the r-GPTSb (*R*^2^ = .07, *p* = .01).

##### Computational measures and self-report

**Figure 2:**
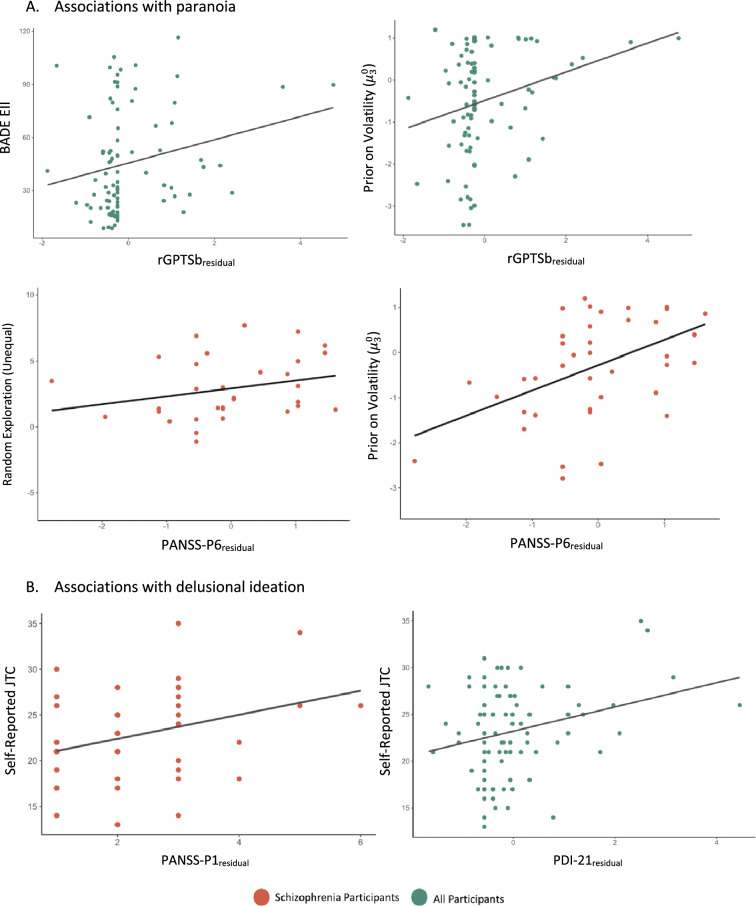
Scatterplots showing linear associations between variables that were significantly associated with (**A**) paranoia and (**B**) delusional ideation in the linear regression models.

In patients, the best model included $\mu_{3}^{0}$ and random exploration (unequal). Only $\mu_{3}^{0}$ ($\beta$ = .48, *p* = .002) and random exploration (unequal) ($\beta$ = .16, *p* = .02) were significantly associated with PANSS-P6 and together explained 40% of the variance (R^2^ = .40, *p* < .001). In the whole sample, group, $\mu_{3}^{0}$, BADE-EII, and random exploration (both equal and unequal) were retained in the best model ; however, only $\mu_{3}^{0}$ ($\beta$ = .29, *p* = .003) and BADE-EII ($\beta$ = 0.012, *p* = .006) were significantly, independently associated with paranoia (Fig. 2A). Together they explained 15% of the variance in r-GPTSb (R^2^ = 0.15, *p* = .004).

#### Delusional ideation—controlling for paranoia

##### Behavioral measures and self-report

In patients, the LASSO regression did not retain any variables, suggesting that none were a good enough predictor of PANSS-P1 in schizophrenia. Across the whole sample, the best model included group, self-reported JTC, BADE and BCIS. Only group ($\beta$ = .46, *p* = .03) and self-reported JTC ($\beta$ = .06, *p* = .004) were significantly associated with PDI-21 and explained 14% of the variance (R^2^ = .14, *p* = .002).

Computational measures and self-report: In patients alone and across the whole sample, the best model for predicting PANSS-P1 and PDI-21 included only self-reported JTC (patients alone: $\beta$ = .09, *p* = .03; whole sample: ($\beta$ = .05, *p* = .04) (Fig. 2B). In patients, self-reported JTC explained 14% variance in PANSS-P1 (R^2^ = .14, *p* = .03). In all participants, it explained 6% variance in PDI-21 (R^2^ = .06, *p* = .04).

#### Additional analyses

##### Raw delusional ideation scores

Results were also examined for regression models predicting paranoia scores without controlling for delusional ideation, and vice versa. These results are presented in Supplemental Table 2 and demonstrated the same pattern of results. The primary difference was that BADE-EII was more strongly associated with both rGPTSb and PDI-21, than was observed in the original models.

##### Including cognitive ability (SCIP) in the regression models

Associations between cognitive ability and decision-making variables are included in Table 2, demonstrating small-to-medium effects. Cognitive ability was measured using a brief neuropsychological assessment of verbal memory (immediate and delayed), verbal fluency, and working memory, called the Screen of Cognitive Impairment in Psychiatry (SCIP)^39^. A z-score was calculated for the total scores (SCIP), based on published norms. This provided a brief, overall assessment of neuropsychological performance. When included in each model, SCIP was never retained after a LASSO procedure was applied. Therefore, all regression models as reported in the section above, and in Table 3, remained unchanged with the inclusion of SCIP, which was shrunk to zero in all models, without impacting the predictors that were retained.

##### Spearman’s correlations

In Table 4, the non-parametric Spearman’s correlation coefficients between delusions and significant variables from the linear regression model are presented.

**Table 4:** Spearman’s ρ correlations reflecting significant associations in a linear model.

	Win Switch rate	Self-reported JTC	$\mu_{3}^{0}$	Random exploration (unequal)	BADE-EII
PANSS-P6_resid_	.35 (.02)	–	.47 (.002)	.35 (.05)	–
r-GPTSb_resid_	.26 (.02)	–	.30 (.006)	–	.27 (.01)
PANSS-P1_resid_	–	.19 (.24)	–	–	–
PDI-21_resid_	–	.23 (.03)	–	–	–

Correlation coefficient (*p*-value). *BADE EII* Bias against disconfirmatory evidence—evidence integration impairment, *P1* Positive and negative syndrome scale positive symptom item 1, *P6* Positive and negative syndrome scale positive symptom item 6, *PDI-21* Peters delusions inventory 21-item, *R-GPTS-b* Revised Green et al. Paranoid thoughts scale. “Resid” indicates that these are residualized scores, in accordance with the linear models. PANSS-P6 controlling for PANSS-P1 and r-GPTSb controlling for PDI-21 (and vice versa).

### Exploratory associations between computational measures and cognitive biases

As this is one of the first examinations of intercorrelations between computational parameters and cognitive biases, we chose to present a broader set of correlations in this dataset that can be followed-up on in larger, subsequent studies. We were particularly interested in relationships between aspects of tasks that measure learning (something that cannot be gleaned from the self-reported JTC measure).

The following are notable associations that, while they did not survive multiple comparisons corrections for the number of tests performed, still merit continued investigation: (1) meta-volatility from the reversal learning task ($\omega_{3}$) was positively related to initial learning rate from the explore/exploit task (ρ = .28, *p* = .02), indicating some convergence in learning parameters across tasks and models; (2) $\omega_{3}$ was negatively associated with BADE-EII (ρ = − 0.32, *p* = .003), suggesting that slower evolution of learning about task volatility (meta-volatility) relates with worse evidence integration during BADE (as hypothesized here^19^), (3) WSR on the reversal learning task was associated with greater decision noise during explore/exploit for equal trials at Horizon 1 (ρ = .44, *p* < .001) and unequal trials at Horizon 6 (ρ = .31, *p* = .01). Decision noise during Horizon 1 equal trials was also significantly associated with $\mu_{3}^{0}$ (ρ = .31, *p* = .008). This may indicate that WSR during reversal learning, and prior belief about volatility, are related to a similar decision-making aspect as random exploration.

Full presentation of correlations between cognitive and clinical variables are presented in Supplemental Table S1.

## Discussion

The current study extends previous findings by examining associations between a variety of decision-making variables, paranoia, and delusional ideation in a single sample of healthy participants and individuals with a schizophrenia-spectrum disorder. Of the cognitive variables, only win-switch rate was different between the groups. Paranoia was best explained by prior beliefs that reward probabilities were volatile and uncertain (motivating greater win-switch rate, $\mu_{3}^{0}$, and random exploration) and poor evidence integration, whereas other themes of delusional ideation were most associated with a self-reported tendency to jump to conclusions. In patients, the computational parameters $\mu_{3}^{0}$ and random exploration explained twice as much variance in persecutory delusion severity as win-switch rate, suggesting that computational modeling may offer more precise estimation of paranoia-relevant decision-making behavior in schizophrenia. Finally, exploratory analyses revealed intriguing associations between: (1) decision noise in the explore/exploit task and both win-switch rate and prior beliefs about volatility in the reversal learning task, as well as (2) between BADE evidence integration impairment and both meta-volatility during reversal learning and initial learning rate during explore/exploit. These preliminary, exploratory associations are broadly consistent with the idea that having a stronger prior belief that a task environment is volatile leads to more random, exploratory decision-making. This can be maladaptive if the environment is less volatile than expected, or if expectations are not updated appropriately by subsequent observations. These results also suggest that BADE evidence integration impairment may tap into similar learning processes as measured in other tasks possibly reflecting how a cognitive bias (i.e. against disconfirmatory evidence) can contribute to differences in learning that are relevant to delusions.

For decades, abnormal decision-making processes have been hypothesized to contribute to delusional thinking. In their seminal paper, Hemsley and Garety^40^ proposed delusions as a deviation from optimal Bayesian learning^40^, a probability-based framework that explains how beliefs are updated in response to an unexpected sensory experience. They laid out several aspects of learning where alterations could contribute to delusional thinking: hypothesis formation, assessment of probabilities, information search for confirmatory or disconfirmatory evidence, and action (i.e., decision-making). This framework has been foundational to continued efforts in understanding how individual differences in learning, reasoning, and decision-making contribute to delusions. Recently, predictive coding has extended the Bayesian learning hypothesis by grounding it in neurobiology^41^. Learning occurs in response to prediction error—a mismatch between expectation and reality that is signaled by dopaminergic neurons in the midbrain and cascades to areas responsible for the experience of salience (e.g., the insula) and decision-making (e.g., the prefrontal cortex)^42^. As noted by Hemsley and Garety, different aspects of this process may be perturbed in individuals who experience delusions and identifying which ones can help inform targeted interventions.

Of the variables measured and tested in the current study, the most consistent findings were that high levels of expected volatility ($\mu_{3}^{0}$, win-switch rate) were related to paranoid thinking in both schizophrenia and healthy participants, while a self-reported tendency for hasty decision-making (self-reported JTC) related to the endorsement of other types of unusual beliefs (e.g., mind reading, ideas of reference, alien control) across the whole sample. Notably, only win-switch rate was significantly different between patients and controls, yet linear associations both within patients and across all participants were observed with delusional thinking, indicating their importance despite the lack of a group difference. These associations are independent from one another in two ways: (1) when included in the same model, volatility priors only ever related to paranoia, while self-reported JTC only ever related to other types of delusional ideation, and (2) volatility priors related to paranoia (controlling for delusional ideation), while self-reported JTC related to delusional ideation (controlling for paranoia). These findings are some of the first to include both aspects of decision-making in the same model and conclude that they are specific, independent predictors of different aspects of delusional thinking. They suggest that decision-making under volatile environments (e.g., reversal learning) is specifically associated with the belief that others intend you harm (as previously reported^43^);, but that quickly updating beliefs based on little evidence is specifically associated with more “bizarre” experiences, such as feeling as if people can read your mind or thinking people can communicate telepathically.

Despite this seeming independence, it is known that persecutory beliefs often overlap with other types of unusual beliefs^44–46^, making it uncommon for people with a psychotic disorder to experience specific or “monothematic” delusions. It may therefore be that the more complex delusional systems observed in many individuals with schizophrenia are the result of *both* altered decision-making in volatile environments and hasty decision-making. One could further speculate that these two processes feed off one another: a hasty decision begets a greater sense of volatility, possibly leading to an urgent need to explain these experiences. Therefore, while seemingly independent, the dynamic interaction between self-reported JTC and volatility priors is an interesting area for future investigation.

A limitation of the JTC interpretation is our use of self-reported JTC, as opposed to a probabilistic reasoning task. Previous work has validated that self-report JTC on the DACOBS is associated with draws to decision on the beads task^15^, but these are clearly very different forms of measurement. Interestingly, however, task-based JTC behavior may not always be an ideal measure, as research on the relationship between task-based JTC and delusion severity is mixed. Meta-analyses have found elevated task-based JTC in individuals with delusions, but no significant association with delusion severity^28^. Two independent, well-powered clinical trials did not find evidence of elevated task-based JTC in psychotic disorders, nor relationships with delusion severity^47^. They also did not find improvement in task-based JTC with treatment^27^, while others have found *reduced* task-based JTC in schizophrenia^48^. Furthermore, additional factors, such as anxiety^49^ and neurocognitive ability^13^ have been suggested to account for task-based JTC alterations. One of the primary explanations for these disparate findings tends to be the type of task used to measure JTC. Therefore, self-reported JTC may avoid some of these difficulties and still index the core cognitive bias at the heart of the JTC concept: an individual’s hasty willingness to accept beliefs that come into their minds.

Another important set of questions within this study was whether computational parameters explained more variance in delusional thinking than more “standard” (descriptive, model-free) behavioral measures, and whether those parameters were themselves intercorrelated. Regarding the first, we found that twice as much variance in paranoia was explained using $\mu_{3}^{0}$ than win-switch rate. $\mu_{3}^{0}$ is an estimate of a participant’s prior belief that the task environment is volatile. In the hierarchical gaussian filter model, this influences how the participant learns and makes subsequent decisions^32,50,51^. For instance, someone with high $\mu_{3}^{0}$ might expect more frequent reversals and therefore be more likely to attribute a probabilistic loss to a reversal (e.g., reduced lose-stay behavior has been observed in schizophrenia^30^). They may also change their behavior more erratically, for instance, switching decks even after a win (elevated win-switch rate). $\mu_{3}^{0}$ may therefore better reflect the overarching dynamics that a volatility prior has on learning and decision-making, which is not fully captured by a single behavior like win-switch rate, and better explains the experience of paranoia. For example, perhaps the faster belief updating associated with high volatility priors could correspond to a type of “overfitting” issue in paranoia, in which random coincidences in observed events are assumed to require hidden explanations.

Furthermore, these data replicate a prior report in schizophrenia^29^ that greater random exploration and lower initial learning rate are associated with positive symptoms; they further suggest that these relationships are stronger with paranoia than unusual thought content. In the current sample, post-hoc exploratory correlations revealed that the relationship with paranoia and random exploration was driven by a tendency to choose the least rewarding option during Horizon 6 when there was unequal information. This means that those who were more paranoid were more likely to explore the task environment than exploit it. In addition, random exploration and priors on volatility were significant independent predictors of paranoia in patients, together explaining 40% of the variance. When considered together, this may be interpreted as follows: individuals who have a more paranoid thinking style hold a stronger prior belief that the environment is unstable, possibly leading to greater willingness to explore the environment in order to manage or anticipate expected change. While speculative, these data offer an intriguing window into how different computational aspects of decision-making can provide insights into clinical phenomena^14,52^.

Finally, the exploratory finding of a relationship between learning rate for volatility (meta-volatility) from the reversal learning task ($\omega_{3}$) and both initial learning rate on the Horizon task and BADE evidence integration impairment (EII) is intriguing. The BADE-EII variable is relatively new variable for analyzing BADE data and was recently shown to be associated with delusional ideation and conspiracy beliefs in the general population^53^. The calculation of BADE-EII is based on the average plausibility rating for implausible items, but it does not take into account *change* in rated plausibility of that item after new evidence is presented. Associations with learning rate suggest that indexing that change through computational modeling could be a fruitful avenue for understanding this reasoning bias and how it relates to other aspects of decision-making.

The consistency we observed in computational parameter estimates across multiple tasks holds promise for the broader project of computational phenotyping in computational psychiatry^54^. That said, it is important to note that, from a theoretical perspective, not all relationships between parameters were in expected directions, and some parameters were not associated with other measures as might have been expected. For example, initial learning rates in the Horizon task were lower in those with prior expectations favoring high volatility in the reversal learning task (and with fewer lose-stays). A-priori, one might expect the opposite (assuming such priors are trait factors that generalize across tasks), since higher volatility implies unexpected observations are more likely due to true environmental change than due to noise that should be ignored. Yet, we also observed a negative correlation between prior on volatility and meta-volatility learning rate, which was itself positively associated with Horizon learning rate. Together this may suggest that stronger expectations of volatility inhibit learning from the environment, as one is working to anticipate environmental changes. One might have also expected that jumping to conclusions would be associated with less exploration, but this was not observed (although, as noted above, JTC was here based on self-report as opposed to a more comparable behavioral measure). Future work will need to clarify whether these results replicate in a new sample and how they should best be interpreted.

It is important to acknowledge limitations of the current report. First, this is a relatively small dataset, and even smaller for those with Horizon task data, which can increase the risk of false positives and false negatives^55^, such as our failure to replicate previous differences between HCs and schizophrenia patients in directed exploration. However, similar effect sizes to those observed in much larger studies^29,31,32,53^ increase our confidence in the significant results we observed. Additionally, the third-level volatility parameters within the reversal learning dataset ($\omega_{3}$, and $\mu_{3}^{0}$) did not demonstrate robust recoverability in simulation (Fig. S1), consistent with previous reports^32^. This may impact confidence on the observed relationships with these third-level parameters. Despite this, clinical relevance of volatility-related belief updating, as measured by third-level of the HGF, continue to be replicated across independent samples (for review, see^56^). Therefore, while we acknowledge this limitation, we hope the findings reported here can add to this growing literature and put it into context with other behavioral and computational variables considered relevant to delusions. The study was conducted virtually, and symptom severity was relatively low in our schizophrenia sample. This may have made it more difficult to detect relationships with clinical symptoms, although prior reports have suggested that clinical assessments can be conducted validly through a virtual platform^56,57^. Finally, as previously noted, JTC was assessed using a self-report, while the other decision-making measures (aside from cognitive insight) were measured through a cognitive task. This limits our ability to make strong conclusions about cognitive processes of jumping to conclusions (i.e., we could not model JTC task data to assess belief instability or stochasticity as has been previously done^9,14^). In addition, as with other self-report measures, it was vulnerabile to socially desirable responding, which may have impacted our findings. Convergence of associations between self-reported JTC and both clinical and self-reported unusual thought content provide some support that our findings were not due to a common reporting bias across measures. Future studies will further explore these dynamics by measuring JTC with a cognitive task.

In summary, we present intercorrelations between decision-making variables collected across five different measures (three tasks, two self-reports) and assess their relative and specific contributions to both paranoia and unusual thought content, in schizophrenia and healthy participants. We observed that decision-making guided by volatility priors and behavioral variability is most related to paranoia, and that self-reported hasty decision-making is most related to other themes of delusional ideation. Computational parameters were better predictors of paranoia in schizophrenia than more standard behavioral measures. Finally, computational model parameters from different cognitive tasks that index learning rates were intercorrelated, lending validity to the interpretation of these measurements across tasks in individuals with schizophrenia and supporting future work on computational phenotyping.

## Methods

### Participants

Individuals aged 18–55 were identified from the Vanderbilt University Medical Center (VUMC) Psychotic Disorders Program. Diagnoses were confirmed by a Structured Clinical Interview of the DSM-IV-TR or DSM-5 (SCID)^58^ completed by a trained rater and signed off in a consensus meeting. Forty-five (45) individuals with a schizophrenia-spectrum disorder (17 schizophrenia, 17 schizophreniform, 7 schizoaffective, 1 psychotic disorder not otherwise specified (NOS)) and 48 healthy control participants with no psychiatric history were recruited. All participants were free of major physical or neurological illness, active substance use disorder, and significant head injury, and had an estimated IQ of > 79. Healthy controls did not have a first-degree relative with a psychotic disorder or any current psychotropic medication use. Study protocol was approved by the Vanderbilt University Institutional Review Board (IRB) in accordance with the relevant guidelines and regulations/ethical principles of the Declaration of Helsinki. Informed consent was provided by all research participants before study participation. All methods were performed in accordance with the guidelines and regulations for human subjects research.

The study was conducted virtually with two-way video and audio and screen sharing for 91% of participants. For those without access to a laptop (9%) the study was conducted in person. As previously reported^30^, multivariate outliers were identified using Mahalanobis Distance calculations. Five multivariate outliers were identified and excluded from further analysis (2 healthy and 3 schizophrenia participants), in addition to 1 healthy participant who was prescribed a psychotropic medication at the time of the study. In addition, explore/exploit (Horizon) task results were not available for 15 participants (5 controls, 10 schizophrenia) due to incomplete data (1 control, 3 schizophrenia), falling asleep during the task (1 schizophrenia), or difficulties with their computer/internet connection during the task (4 controls, 6 schizophrenia).

**Figure 3:**
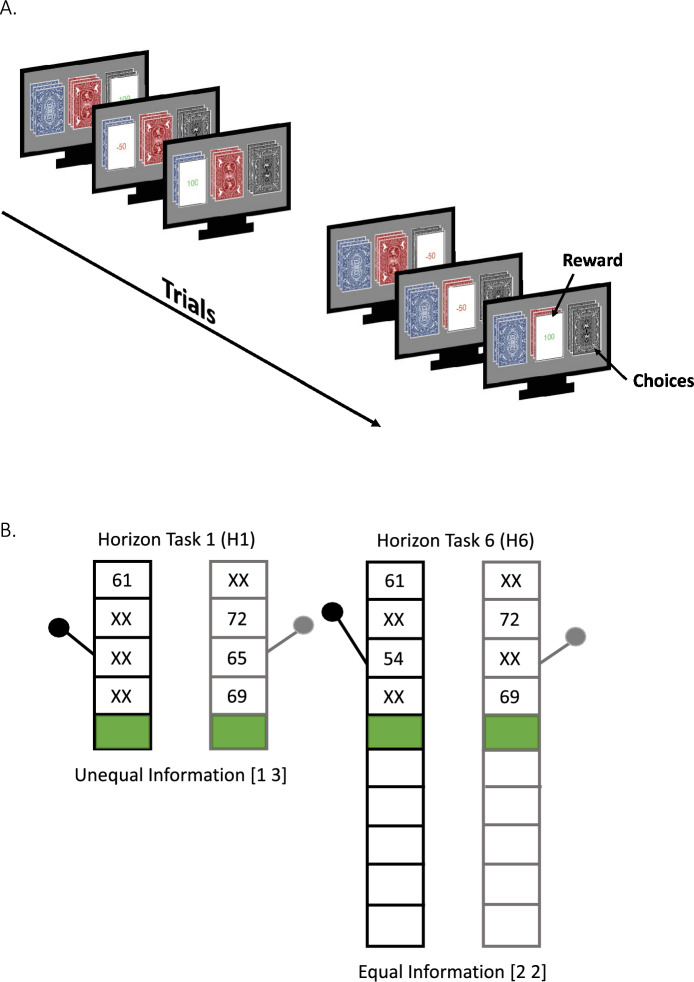
(**A**) 3-option probabilistic reversal learning task (3PRL). Three decks of cards are presented and the participant is told to find the “best deck” that wins points the most often. They are also told that at some point in the experiment, the best deck might change, in which case they should try and find the new best deck. The task consisted of four blocks of 40 trials each. The 1st and 2nd blocks had reward contingencies of 90%–50%–10% reward and the 3rd and 4th blocks had reward contingencies of 80%–40%–20%. Participants were not told that the reward contingencies had changed. (**B**) Horizon task. Participants completed 80 self-paced games in which they had to choose between virtual slot machines, with the goal of earning the most points. The average reward provided by each slot machine was sampled from a Gaussian distribution and was unknown to the participant. They had to be learn the reward given by the machines through a combination of forced choice and free choice trials. The number of free choices was either 1 (Horizon 1) or 6 (Horizon 6), and manipulated how valuable it was to explore. In Horizon 1, with only one free choice, exploration has no value because no additional choices will be made in the future. In Horizon 6, exploring may be initially beneficial, in order to gain information that guides decision-making. In an unequal condition [1 3], the participant has unequal information from each machine, and in an equal condition [2 2], participant see two pieces of information from each machine.

The final sample for behavioral data therefore includes 42 schizophrenia and 45 healthy participants (Table 1). Computational modeling analyses were conducted in the subset of individuals who completed the Horizon task: 32 schizophrenia and 40 healthy participants (Fig. 3).

### Self-report and task-based measures

#### Self-reported jumping to conclusions (JTC)

Jumping to conclusions (JTC) was assessed using the Davos Assessment of Cognitive Biases Scale (DACOBS)^15^, a self-report measure of reasoning biases common to the experience of psychosis. While JTC was measured using self-report, not a cognitive task, JTC scores on the DACOBS have been previously validated as significantly associated with performance on the Beads Task in schizophrenia^15^, allowing for an estimate of the JTC reasoning bias. JTC is measured using six self-report items that include “I make decisions faster than other people”, “I quickly find evidence to support my beliefs”, “The right conclusion often pops in my mind”, “The first thoughts are the right ones”, “I don’t need long to reach a conclusion”, “I don’t need to evaluate all the facts to reach a conclusion”, and “I don’t need to consider alternatives when making a decision”. Each item is scored on a Likert scale from 1 to 7 (strongly disagree to strongly agree). A sum total of the six items (range 6–35) was used as a measure of JTC.

#### Self-reported cognitive insight

Cognitive insight was measured using the Beck Cognitive Insight Scale (BCIS;^22^). The BCIS is a 14-item self-report scale with nine items measuring self-reflectiveness and five items measuring self-certainty. A composite score was calculated (self-reflectiveness minus self-certainty) to reflect cognitive insight—how reflective the individual self-reports that they are, adjusting for their self-reported level of certainty.

#### Task-based bias against disconfirmatory evidence (BADE)

BADE was assessed using an abbreviated version of the commonly employed task developed by Woodward and colleagues^20^. This task includes 12 scenarios and four explanations for each scenario. Explanations are presented three times and the participant must rate how “possible” each explanation is using a scroll bar from “Not Possible” to “Very Possible”. After each rating, additional information about the scenario is provided to the participant. Of the four potential explanations, two are Lure explanations (Lure-A/Lure-B), which start off as possible but become less likely as more information about the scenario is shared. One explanation is True (moderately possible initially and then the most possible once all information is learned). One explanation is Absurd (consistently implausible).

BADE data were scored after the method in Bronstein and Cannon^21^. Specifically, evidence integration impairment (EII) was scored as follows: (Absurd 1 + Absurd 2 + Absurd 3) /3 + (Lure-A 3 + Lure-B 3)/2) for EII. EII scores how the individual rated the explanations that should have been least plausible, given the evidence provided to the participant. Higher EII reflects higher plausibility given to implausible explanations.

#### Task-based probabilistic reversal learning

Reversal learning from this dataset has been described previously^30^. Briefly, belief updating was measured using a 3-option probabilistic reversal learning (3-PRL) task, during which participants were presented with three decks of cards on a computer screen and told that each deck includes both winning (+ 100 points) and losing (− 50 points) cards, but that some decks win more often than others (Fig. 1a). They were instructed to find the ‘best’ deck—the one with the highest probability of reward. They were also told that the best deck might change, whereupon they should try and find the new best deck. Participants’ overarching goal was to win the most points they could.

Unbeknownst to the participant, the decks yielded 90%, 50%, and 10% reward for blocks 1 and 2 (80 trials) and then these contingencies changed at the start of block 3–80%, 40%, 20% (“contingency transition”). Additionally, the best deck changed when a participant selected it in nine out of ten consecutive trials (“reversal events”). The goal of this task structure was to make it more difficult for participants to discern whether a loss was due to probabilistic noise or due to the best deck changing^32^.

Belief updating was measured behaviorally as win-switch rate, which reflects the number of times a participant switched decks after receiving positive feedback (+ 100 points), divided by the number of trials in which they received positive feedback. We also measured lose-stay rate, which measures an individual’s willingness to stick with a losing deck, presumably because it may still represent the “best” deck.

Computational modeling was also conducted on data from the 3-PRL task using the Hierarchical Gaussian Filter toolbox^50,51^ in MATLAB 2020b (MathWorks, Natick, MA) as previously described^30^. Belief updating parameters were calculated for the 90–50–10 reward contingencies (1st and 2nd blocks) and the 80–40–20 contingencies (3rd and 4th blocks). Participant data was entered as separate column vectors for each block, modeling deck choice (deck 1,2, or 3) and outcome (win or loss), using an autoregressive 3-level HGF multi-arm bandit configuration for the perceptual model, paired with a Softmax decision model, in which inverse temperature ($\beta$) is inversely proportional to volatility estimates ($\mu_{3}^{0} )$. Belief updating trajectories are represented as probability distributions that encode belief content and uncertainty and were specific to each participant, due to the probabilistic and performance-dependent nature of the task. Analyses were completed using scripts that have been previously reported and shared^31^: https://github.com/psuthaharan/covid19paranoia. Previous work has tested the propriety of this 3-level HGF through simulations and comparison with alternative models^32^. Simulation recovers group differences between elevated and low paranoia better than simpler models, suggesting its appropriateness for examining relationships with paranoia. Model fit was estimated using Bayesian Information Criterion (BIC) and model fit did not differ between schizophrenia and healthy comparison participants (t(89) = − .73, *p* = .47).

The computational model yields the following parameters in a 3-level hierarchical model: $\sigma$_2_, $\sigma$_3_, µ_2,_ $\varphi$_2,_ $\varphi$_3,_ $\omega_{2}$, $\kappa$, $\omega_{3}$, and $\mu_{3}^{0}$. Of those, the last four parameters were of particular interest, based on their measurement of cognitive processes of interest. Level 1 is trial-by-trial perception of win or loss feedback, Level 2 is the stimulus-outcome associations (e.g. 90–50–10) and Level 3 is the perception of the overall reward contingency context (i.e., how much it is changing; volatility). At Level 2, $\omega_{2}$ reflects a baseline (or stable component) of volatility, reflecting the dispersion of the random walk at Level 2. $\kappa$ reflects how sensitive the individual is to unexpected changes (sensitivity to volatility), and is the overall impact of Level 3 (volatility estimates) on belief updating at Level 2 (deck-reward associations). At Level 3, $\omega_{3}$ reflects meta-volatility, which is the volatility learning rate for the updating of beliefs regarding how volatile the task environment is. This parameter is based on the dispersion of the random walk at Level 3. The primary computational parameter of interest, however, was $\mu_{3}^{0}$, which estimates the initial (prior) belief about volatility at Level 3 of the model. This parameter estimates the participant’s belief about how volatile (changing, unstable) the task environment is at the beginning of the experiment. Higher $\mu_{3}^{0}$ values indicate a stronger expectation that the contingencies in the experiment will shift erratically. As previously published, although other parameters can be modeled from this data, $\mu_{3}^{0}$ shows the strongest relationship with paranoia^30,31^. We therefore only included $\mu_{3}^{0}$ as a dependent variable in our models, but present a full picture of relationships between these computational variables and other decision-making measures in the Supplement. In addition, we examined the recoverability of these HGF parameters using simulations, which are presented in the Supplement.

#### Task-based explore/exploit decision-making

Participants completed the Horizon Task^33^ (Fig. 3b) a 2-option reward learning task designed to create an explore/exploit dilemma—participants must choose between exploiting options with known reward probabilities/magnitudes versus exploring options with unknown reward probabilities/magnitudes (i.e., which could be better than known options). A computational model can be fit to behavior and provide estimates of parameter values associated with decision noise, the magnitude of an information bonus assigned to actions with more uncertain outcomes, and learning rate. Learning rate indexes how quickly the individual updates their beliefs about expected outcomes of each option after being presented with new observations. Theoretically, a slower learning rate is often assumed to reflect an implicit prior belief that reward probabilities are stable and/or that reward outcomes have high variance around the mean (i.e., in both cases, beliefs should not change very much after single unexpected observations). In uncertain situations, it can often be adaptive to start with a high learning rate, which slowly reduces over time as confidence in expected outcomes increases.

In this task, participants played a series of 80 games in which they had to choose between two slot machines, with the goal of earning as many points as possible. The first four choices in each game are forced choice. In some games they must sample two outcomes from each machine (equal information condition). In other games, they must sample three outcomes from one machine and one outcome from the other (unequal information condition). Directed exploration then corresponds to choosing the machine with fewer sampled outcomes on the first free choice (choice 5). Half the games have only one free choice (Horizon 1; H1), while half have six free choices (Horizon 6; H6), which is known to increase directed exploration. It also increases random exploration, which corresponds to choosing the option with the lower observed mean. The variance in reward values across all games/choices is the same. The difference in reward means between options is systematically varied between − 30 and + 30 for the left option with respect to the right option.

The model we used has been described in detail elsewhere^59^. It combines a learning model applied to the four forced choices and a decision model applied to the first free (fifth) choice. The learning model uses a Kalman filter for learning the mean reward value for each option. Expected mean reward value $R$ for each option $i$ on trial $t$ is updated based on the following equation, based on a prediction error with respect to observed reward $r_{t}$:

$$ R_{t + 1}^{i} = R_{t}^{i} + \alpha_{t}^{i} \left( {r_{t} - R_{t}^{i} } \right) $$

Expected mean reward values are not updated for unplayed options. The learning rate $\alpha$ is based on the standard deviation in the fixed Gaussian distribution from which rewards in each option are generated ($\sigma_{r}$) and how the uncertainty in that estimate changes over time ($\sigma_{t + 1}^{i}$):

$$ \alpha_{t}^{i} = \frac{{\left( {\sigma_{t + 1}^{i} } \right)^{2} }}{{\sigma_{r} }} $$

This learning rate can then be updated over time, while incorporating a possible drift in the mean reward over time (although the true reward means are stable). This drift is based on a Gaussian random walk with a mean of 0 and standard deviation $\sigma_{d}$:

$$ \alpha_{d} = \frac{{\sigma_{d}^{2} }}{{\sigma_{r}^{2} }} + 1 $$

$$ \frac{1}{{\alpha_{t}^{i} }} = \frac{1}{{\alpha_{t - 1}^{i} + \alpha_{d} }} + 1 $$

As done previously, we use behavioral data to fit the initial expected reward mean $R_{0}$, as well as the initial $\alpha_{1}$ asymptotic $\alpha_{\infty }$ learning rates:

$$ \alpha_{1} = \frac{{\sigma_{0}^{2} + \sigma_{d}^{2} }}{{\sigma_{r}^{2} }} $$

$$ \alpha_{\infty } = \frac{1}{2}\left( { - \alpha_{d} + \sqrt {\alpha_{d}^{2} + 4\alpha_{d} } } \right) $$

It follows that learning rate will be higher if initial uncertainty (or, equivalently, expected outcome noise) is higher and/or if the expected instability in reward means is higher).

The decision model was based on a simple logistic choice rule based on the learned reward values:

$$ p\left( {choose right} \right) = \frac{1}{{1 + {\text{exp}}\left( {\frac{{{\Delta }R + A{\Delta I} + {\text{B}}}}{\sigma }} \right)}} $$

here ${\Delta }R = R_{t}^{left} - R_{t}^{right}$, ${\Delta }I = + 1$ when the left option was more informative and $- 1$ when the right option was more informative (and $0$ in equal information conditions), ${\text{A}}$ is an information bonus parameter, ${\text{B}}$ is a spatial bias parameter accounting for a preference to choose one side vs. the other, and $\sigma$ is the decision noise. The information bonus, decision noise, and spatial bias parameters were fit to participant data separately for the H1 and H6 conditions. This allows replication of previously results in which both information bonus and decision noise are greater in H6 than H1—reflecting directed and random exploration, respectively.

Each of the model parameters ($R_{0}$, $\alpha_{1}$, $\alpha_{\infty }$; and $A$*,* $B$*,* and $\sigma$ for the different horizon and equal vs. unequal information conditions) were estimated using a hierarchical Bayesian approach. This approach assumes that each parameter for each participant is sampled from a group-level prior distribution. The parameters for these group-level distributions were estimated using a Markov Chain Monte Carlo (MCMC) sampling algorithm, implemented in the JAGS package^60^ in Matlab (psiexp.ss.uci.edu/research/programs_data/jags/). The group-level priors, and all other procedures for parameter estimation, were identical to (and run using the same Matlab code) as done in previous studies^59^. The reader is referred to this prior work for more technical details.

For inclusion in the main analyses, random exploration values (under unequal and equal information conditions), defined as H6 $\sigma$ values minus H1 $\sigma$ values, were selected. This was based on prior work demonstrating that positive symptoms of schizophrenia were associated with random exploration during the same Horizon explore/exploit task^29^. Although learning rate was also found to be related to positive symptoms in that dataset, random exploration was chosen as a more informative index of explore/exploit decision-making specifically. Directed exploration was calculated as H6 $A$ minus H1 $A$ values. While not a primary aim of this paper, we did examine whether directed exploration was lower in schizophrenia patients than healthy controls, as this was observed in one previous study^29^.

#### Task-based cognitive ability

Cognitive ability was measured using the Screen for Cognitive Impairment in Psychiatry (SCIP;^39^). The SCIP includes measures of verbal memory (immediate and delayed), verbal fluency, and working memory (note: the processing speed section of the SCIP was not completed as the majority of study visits were conducted virtually). SCIP subtest raw scores were converted to z-scores using normative data and averaged to create a composite z-score^39^. The SCIP has been shown to be a reliable and valid measure of cognitive ability in psychotic disorders^61^.

### Delusion assessments

Severity of paranoid and delusional thinking was measured in schizophrenia participants via clinical interview and via self-report in all participants.

In the schizophrenia group, the Positive and Negative Syndrome Scale (PANSS)^36^ was used to assess both unusual thought content (P1) and suspiciousness/persecution (P6). These items were selected due to their specific assessment of delusional ideation, as opposed to overall “positive symptoms”, which would include aspects of hallucinations, disorganization and excitement not being investigated. Each item is rated on a 7-point Likert scale from Absent to Extreme. Regardless of current PANSS score, all but one schizophrenia participants had experienced a clinically-significant delusion at some point in the history of their illness. Of the 42 participants, 36 (86%) had experienced a persecutory delusion at some point. Of those, only three participants had experienced *only* a persecutory delusion. Six participants had experienced *only* unusual thought content (e.g., grandiose, passivity delusion, religious, or bizarre delusion). All other schizophrenia participants had a history of multiple delusional themes including persecutory and unusual thought content.

Self-reported paranoia and unusual thought content were measured across all participants using the revised Green et al*.* Paranoid Thoughts Scale (R-GPTS)^34^, which includes a 10-item scale measuring persecutory ideation (r-GPTSb) and the Peters et al*.* Delusions Inventory (PDI-21)^35^, which assesses endorsement (yes/no), level of distress, conviction, and preoccupation related to a variety of delusional beliefs. All PDI-21 items were summed to create a total delusional ideation score.

### Data analysis

Group differences in cognitive variables were assessed in independent samples Mann–Whitney U Test. Zero-order Spearman’s rho correlations were conducted to examine the inter-relationship between reasoning biases and decision-making variables.

In order to determine the proportion of variance explained in delusional thinking by cognitive variables, we conducted four linear regressions (as has been done previously^62^, predicting (1) paranoia in patients (PANSS-P6), (2) delusions in patients (PANSS-P1), (3) paranoia across all individuals (R-GPTS-b), and (4) delusional ideation across all participants (PDI-21 total score). Regression controlled for age, gender, and group (when all participants included). Of note, symptom ratings were residuals, controlling for the other delusional rating scale: PANSS-P6 controlling for PANSS-P1 in patients, GPTS-b controlling for PDI-21 total (and vice versa). This allowed us to try and isolate severity of paranoia independent of overall unusual thought content and isolate overall delusional ideation independent of paranoia. Variance explained was estimated using R^2^.

To help determine specificity of the relationship between cognitive variables and delusional thinking, and minimize the impact of multicollinearity and overfitting amongst our dependent variables, a least absolute shrinkage and selector operator (LASSO) regularization method was used prior to conducting the linear regressions described above. This method can aid in variable selection, by shrinking small coefficients to zero by adding a penalty term in the process of model estimation^38^. The process was conducted in R using glmnet^37^. The variables retained in the model following the LASSO technique indicated those variables that had a significant, independent relationship with delusional thinking. Only those predictor variables were then retained in the linear regression model, which was analyzed in R using lm.

Prior to conducting linear regressions, all dependent variables (except age, gender and group) underwent log transformation using the optLog package in R to minimize the impact of skew.

## Supplementary Information

Supplementary Information.

## Data Availability

De-identified datasets analyzed during the current study have been made available on Github for download at https://github.com/JuliaSheffield/CogMech_delusions.
