# Supplementary material for: Relationships between cognitive biases, decision-making, and delusions
Source: Sci Rep. 2023 Jun 10;13:9485. doi: 10.1038/s41598-023-36526-1 (PMC10257713; doi:10.1038/s41598-023-36526-1)
Supplement: Supplementary file 1 — Supplementary Information. [file 41598_2023_36526_MOESM1_ESM.docx]

**Supplemental Materials**

Table S1a: Associations between all computational and behavioral variables from reversal learning and explore/exploit tasks

|  | **Reaction Time** | **Win-Switch Rate** | **Lose-Stay Rate** | $\boldsymbol{\mu}_{\boldsymbol{3}}^{\boldsymbol{0}}$ | $\boldsymbol{\kappa}$ | $\boldsymbol{\omega}_{\boldsymbol{2}}$ | $\boldsymbol{\omega}_{\boldsymbol{3}}$ |
| --- | --- | --- | --- | --- | --- | --- | --- |
| **Learning Rate (start)** | -.05 | **-.42 (<.001)** | .15 | **-.28 (.02)** | -.04 | .05 | **.28 (.02)** |
| **Learning Rate (asymptotic)** | .08 | .05 | -.22 | .10 | -.07 | .112 | .05 |
| **Directed Exploration** | -.10 | -.02 | .22 | -.21 | .16 | -.23 | .10 |
| **Random Exploration (equal)** | **-.35 (.003)** | **-.25 (.03)** | **.35 (.002)** | **-.24 (.047)** | .07 | -.16 | -.03 |
| **Random Exploration (unequal)** | -.07 | .09 | .11 | .09 | .18 | -.09 | -.14 |
| **Info Bonus H1** | .19 | .09 | .05 | -.08 | .06 | -.08 | .09 |
| **Info Bonus H6** | -.08 | .08 | .19 | -.13 | .16 | **-.28 (.02)** | .02 |
| **Decision Noise H1 [1 3]** | -.03 | **.24 (.04)** | -.10 | .15 | -.00 | -.09 | **-.24 (.04)** |
| **Decision Noise H1 [2 2]** | .16 | **.44 (<.001)** | **-.27 (.02)** | **.31 (.008)** | .12 | -.04 | -.22 |
| **Decision Noise H6 [1 3]** | .02 | .31 (.008) | -.03 | .23 | .18 | -.08 | -.18 |
| **Decision Noise H6 [2 2]** | -.10 | .20 | -.03 | .15 | .15 | -.17 | **-.26 (.03)** |

Table S1b: Associations between explore/exploit variables, reasoning biases and cognitive data

|  | **Age** | **WTAR (Premorbid IQ)** | **SCIP (cognitive ability)** | **Self-reported JTC** | **BADE-EII** | **BCIS** |
| --- | --- | --- | --- | --- | --- | --- |
| **Learning Rate (start)** | .15 | **.45 (<.001)** | **.40 (<.001)** | -.15 | .01 | .05 |
| **Learning Rate (asymptotic)** | -.12 | -.02 | .17 | -.15 | -.05 | **-.24 (.04)** |
| **Directed Exploration** | -.09 | .18 | .23 | -.03 | -.08 | .04 |
| **Random Exploration (equal)** | -.03 | .15 | .09 | .14 | .00 | .11 |
| **Random Exploration (unequal)** | -.18 | .06 | -.13 | .23 | **.25 (.04)** | .21 |
| **Info Bonus H1** | -.04 | .09 | .13 | .18 | .03 | -.07 |
| **Info Bonus H6** | -.09 | .07 | .15 | .04 | -.03 | .04 |
| **Decision Noise H1 [1 3]** | -.14 | **-.33 (.005)** | **-.28 (.02)** | -.05 | -.00 | -.01 |
| **Decision Noise H1 [2 2]** | -.15 | **-.44 (<.001)** | **-.28 (.02)** | -.11 | .16 | -.05 |
| **Decision Noise H6 [1 3]** | **-.27 (.02)** | -.23 | **-.37 (.001)** | .11 | .20 | **.23 (.049)** |
| **Decision Noise H6 [2 2]** | **-.24 (.046)** | **-.30 (.01)** | **-.25 (.04)** | -.01 | -.09 | .00 |

Table S1c: Associations between reversal learning variables, reasoning biases and cognitive data

|  | **Age** | **WTAR (Premorbid IQ)** | **SCIP (cognitive ability)** | **Self-Reported JTC** | **BADE-EII** | **BCIS** |
| --- | --- | --- | --- | --- | --- | --- |
| **Reaction Time** | .13 | **-.38 (<.001)** | **-.38 (<.001)** | -.11 | .17 | -.07 |
| **Win-Switch Rate** | **-.25 (.02)** | **-.43 (<.001)** | **-.36 (<.001)** | .06 | .17 | -.10 |
| **Lose-Stay Rate** | .02 | **.27 (.01)** | **.28 (.01)** | .13 | .18 | .02 |
| $\boldsymbol{\mu}_{\boldsymbol{3}}^{\boldsymbol{0}}$ | -.08 | **-.31 (.004)** | **-.23 (.03)** | -.18 | .04 | -.07 |
| $\boldsymbol{\kappa}$ | **-.24 (.02)** | -.07 | .04 | -.01 | .18 | -.03 |
| $\boldsymbol{\omega}_{\boldsymbol{2}}$ | .11 | -.02 | -.09 | -.05 | -.14 | .01 |
| $\boldsymbol{\omega}_{\boldsymbol{3}}$ | .03 | .12 | .11 | -.01 | **-.32 (.003)** | -.06 |

Table S1d: Intercorrelations between Horizon Task (explore/exploit) variables

|  | Learning Rate (start) | Learning Rate (asymptotic) | Directed Exploration | Random Exploration (equal) | Random Exploration (unequal) | Info Bonus H1 | Info Bonus H6 | Decision Noise H1 [1 3] | Decision Noise H6 [1 3] | Decision Noise H1 [2 2] | Decision Noise H6 [2 2] |
| --- | --- | --- | --- | --- | --- | --- | --- | --- | --- | --- | --- |
| Learning Rate (start) | -- |  |  |  |  |  |  |  |  |  |  |
| Learning Rate (asymptotic) | 0.10 | -- |  |  |  |  |  |  |  |  |  |
| Directed Exploration | -0.07 | 0.07 | -- |  |  |  |  |  |  |  |  |
| Random Exploration (equal) | -0.04 | -0.003 | **0.33**** | -- |  |  |  |  |  |  |  |
| Random Exploration (unequal) | -0.10 | 0.02 | 0.05 | 0.12 | -- |  |  |  |  |  |  |
| Info Bonus H1 | 0.19 | 0.01 | 0.02 | -0.11 | -0.06 | -- |  |  |  |  |  |
| Info Bonus H6 | -0.21 | 0.05 | **0.87*** | **0.28*** | 0.01 | **0.25*** | -- |  |  |  |  |
| Decision Noise H1 [1 3] | -**0.69**** | -0.10 | 0.02 | 0.13 | -0.21 | **-0.41**** | 0.11 | -- |  |  |  |
| Decision Noise H6 [1 3] | **-0.52**** | -0.11 | -0.02 | 0.08 | **0.68**** | **-0.24*** | 0.05 | **0.39**** | -- |  |  |
| Decision Noise H1 [2 2] | **-0.56**** | -0.03 | -0.18 | **-0.37**** | 0.03 | -0.10 | -0.04 | **0.44**** | **0.31**** | -- |  |
| Decision Noise H6 [2 2] | **-0.61**** | 0.02 | 0.21 | **0.56**** | 0.15 | -**0.22*** | **0.29**** | **0.54**** | **0.40**** | **0.37**** | -- |

*p<.05, **p<.01, ***p<.001

Table S1e: Intercorrelations between probabilist reversal learning (PRL) task variables

|  | Reaction Time | Win-Switch Rate | Lose-Stay Rate | $\boldsymbol{\omega}_{\boldsymbol{2}}$ | $\boldsymbol{\kappa}$ | $\boldsymbol{\omega}_{\boldsymbol{3}}$ | $\boldsymbol{\mu}_{\boldsymbol{3}}^{\boldsymbol{0}}$ |
| --- | --- | --- | --- | --- | --- | --- | --- |
| Reaction Time | -- |  |  |  |  |  |  |
| Win-Switch Rate | .43** | -- |  |  |  |  |  |
| Lose-Stay Rate | -.34** | -.22* | -- |  |  |  |  |
| $\boldsymbol{\omega}_{\boldsymbol{2}}$ | .05 | -.32** | -.64** | -- |  |  |  |
| $\boldsymbol{\kappa}$ | .02 | .45** | .42** | -.81** | -- |  |  |
| $\boldsymbol{\omega}_{\boldsymbol{3}}$ | -.22* | -.31** | -.16 | .49** | -.40** | -- |  |
| $\boldsymbol{\mu}_{\boldsymbol{3}}^{\boldsymbol{0}}$ | .37** | .57** | -.28** | -.22* | .44** | -.46** | -- |

*p<.05, **p<.01, ***p<.001

Table S2: Results of LASSO regression using raw paranoia and delusional ideation scores

|  | PANSS-P6 | r-GPTSb | PANSS-P1 | PDI-21 |
| --- | --- | --- | --- | --- |
| Age | . | . | . | . |
| Gender | 0.48 | . | . | . |
| Group | *N/A* | . | *N/A* | 11.25* |
| WSR | 0.27* | 0.74* | . | . |
| BADE-EII | . | 0.03* | . | 0.14 |
| Self-Reported JTC | . | 0.08 | .08* | 1.65** |
| BCIS | . | . | . | . |

|  | PANSS-P6 | r-GPTSb | PANSS-P1 | PDI-21 |
| --- | --- | --- | --- | --- |
| Age | . | . | . | 0.39 |
| Gender | 0.33 | . | . | . |
| Group | *N/A* | . | *N/A* | 10.2 |
| $\boldsymbol{\mu}_{\boldsymbol{3}}^{\boldsymbol{0}}$ | 0.54** | 0.71* | . | . |
| BADE-EII | . | 0.04*** | . | 0.28** |
| Random Exploration (Unequal) | 0.20** | . | . | . |
| Random Exploration (Equal) | . | . | . | . |
| Self-Reported JTC | . | . | . | 1.38* |
| BCIS | . | . | . | 0.81 |

****p<.001, **p<.01, *p<.05*

*PANSS-P6: Suspiciousness/Persecution as measured in schizophrenia; r-GPTSb: self-reported paranoia as measured in all participants; PANSS-P1: Delusions as measured in schizophrenia; PDI-21: delusional ideation as measured in all participants*

|  |
| --- |
|  |
|  |
|  |
|  |
|  |
|  |
|  |
|  |
|  |

Figure S1: simulation and parameter recovery for the Hierarchical Gaussian Filter (HGF)

We performed ten simulations per participant using the participant’s actual data (their choices and the corresponding outcome) and their derived set of perceptual parameters. Simulated data based on these subject parameters were then used to recover parameter estimates. Recovered HGF parameters (10 per participant) were then averaged and correlated with the participant’s actual parameters from their performance on the task. Group differences (healthy versus schizophrenia participants) were also calculated for the recovered parameters. As in the actual data, no group differences in HGF parameters were observed for the recovered data. In terms of correlations, we observed significant positive correlations between recovered and actual parameter estimates for the second-level parameters ($\omega_{2}$(r=.65, p<.001) and $\kappa$ (r=.29, p=.008)) but not third-level parameters ($\mu_{3}^{0}$ (r=-.02, p=.85) and $\omega_{3}$ (r=.21, p=.05)).

Figure S2: Group Differences in self-report and task-based variables

Group differences in cognitive variables. Directed exploration and learning rate were included in order to directly replicate analyses reported by Waltz et al. (2020). *p<.05 using Mann-Whitney U Test.
